# Structural colour from helicoidal cell-wall architecture in fruits of *Margaritaria nobilis*

**DOI:** 10.1098/rsif.2016.0645

**Published:** 2016-11

**Authors:** Silvia Vignolini, Thomas Gregory, Mathias Kolle, Alfie Lethbridge, Edwige Moyroud, Ullrich Steiner, Beverley J. Glover, Peter Vukusic, Paula J. Rudall

**Affiliations:** 1 Chemistry Department, University of Cambridge, Lensfield Road, Cambridge CB2 1EW, UK; 2 Royal Botanic Gardens Kew, Richmond, Surrey TW9 3AB, UK; 3 Thin Film Photonics, School of Physics, Exeter University, Exeter EX4 4QL, UK; 4 Massachusetts Institute of Technology, 77 Massachusetts Avenue, Cambridge, MA 02139-4307, USA; 5 Department of Plant Sciences, University of Cambridge, Downing Street, Cambridge CB2 3EA, UK; 6 Adolphe Merkle Institute, Chemin des Verdiers 4, 1700 Fribourg, Switzerland

**Keywords:** structural colour, helicoidal cell wall, circular dichroism, cellulose, iridescence, natural photonics

## Abstract

The bright and intense blue-green coloration of the fruits of *Margaritaria nobilis* (Phyllanthaceae) was investigated using polarization-resolved spectroscopy and transmission electron microscopy. Optical measurements of freshly collected fruits revealed a strong circularly polarized reflection of the fruit that originates from a cellulose helicoidal cell wall structure in the pericarp cells. Hyperspectral microscopy was used to capture the iridescent effect at the single-cell level.

## 1. Introduction

In some plants, the cell walls of selected tissues exhibit helicoidal architecture, in which multiple adjacent wall layers are composed of aligned cellulose fibrils that rotate along a helical screw [1]. Despite this regular construction, considerable flexibility exists in the dimensions and geometry of the multi-layered structure [2]. In the special case when the dimension of the helicoid, defined by the distance between two planes with closely similar fibril orientation (half of a full 360° rotation, pitch *p*), is comparable to the wavelength of visible light and is constant within the cell wall, these structures are capable of selectively reflecting coloured light that may be polarized. In particular, they reflect circularly polarized light at a wavelength defined by *λ* = *np* (where *n* is the mean refractive index of the medium) and with optical handedness that depends on the handedness of the helicoid [3].

Helicoidal cell-wall architecture has been reported in a broad range of land plants, including mosses, ferns, gymnosperms and angiosperms [2,4], but they are also common in beetle exoskeletons [5]. This apparently complex cell-wall structure occurs in tissues that include thick-walled cells [6], including epidermis, sclerenchyma and xylem and in many different plant organs, including leaves, stems and fruits [1,7–11]. For example, structural colour obtained from helicoidal architecture has been reported in leaves of plants from a range of different habitats [12–15]. However, with a few exceptions (e.g. hazelnut [16], *Pollia* [11,17]), this structure has rarely been studied in fruits and seeds, which often possess thick-walled tissues that are resistant to desiccation. Most fruit colour is produced by pigmentation [18], but a few plant species produce highly metallic and intensely coloured fruits by means of a nanostructured multi-layered cell wall, including the commelinid monocot *Pollia condensata* [11,17] and the rosid eudicot *Margaritaria nobilis* [19,20].

In this paper, we use both polarization-resolved spectroscopy and electron microscopy to present a detailed optical analysis of fresh fruits of *M. nobilis* (Phyllanthaceae), a forest tree from tropical Central and South America. In this species, the fruits possess a green exocarp, which splits after they become detached and fall to the forest floor [19,20]. The remaining exposed inner part of the fruit wall exhibits a metallic greenish-blue colour, particularly in humid environments, that is attractive to birds such as jays and doves [19]. These birds consume the fruits and hence act as dispersal agents. The results obtained here demonstrate that the strong intense coloration of *M. nobilis* fruits is due to a helicoidal cellulose structure in the endocarp cell walls. The optical measurements are confirmed by high-resolution electron microscopy of the tissue showing a Bouligand pattern typical of helicoidal architectures [21].

The fruits of *M. nobilis* are only the second example of a plant species that has been conclusively demonstrated to use helicoidal cell-wall architecture to produce structural colour. The first example was of the fruits of the commelinid monocot *P. condensata* [11,17]. This is a surprising discovery because of the evolutionary distance separating *Margaritaria* and *Pollia*. The use of a cellulose helicoidal architecture to produce colour has clearly evolved independently and convergently in these two species, which are estimated to have diverged over 100 Ma. This finding suggests that helicoidal structures represent a possible strategy for convergent evolution of structural colour in plants.

## 2. Material and methods

### 2.1. Plant material

For optical and microscopic analysis, fresh fruits were collected in Panama under permit *SEX*/*P*-59-13 to Dr Edmund Tanner (issued 23 October 2013 by the Direccion de Areas Protegidas y Vida Silvestre). Fruits were refrigerated and then sent directly to Cambridge, UK. For examination, using light microscopy, scanning electron microscopy (SEM) and transmission electron microscopy (TEM), fruits were also obtained from the Royal Botanic Gardens, Kew, either from alcohol-preserved specimens (collected from Brazil by Milliken in 2011) or dried herbarium specimens from two separate collections, the first collected by Spruce in 1855, and the second collected by Belem and Mendes in 1964.

### 2.2. Microscopy

Optical imaging was performed using a customized Zeiss optical microscope equipped with epi-illumination and a 10× objective. Unpolarized light from a halogen lamp served as illumination for imaging. A polarizer and a quarter-waveplate mounted onto independent motorized rotation stages were selectively inserted into the optical path to perform polarization-resolved imaging.

For SEM imaging, dried fruit material was fractured, mounted on an aluminium stub, coated with platinum using a sputter coater (Quorum Q150T ES) and examined using a Hitachi S-4700 SEM at 2 kV.

For TEM imaging, fruits were cut into small fragments and fixed in 3% phosphate-buffered glutaraldehyde followed by immersion in 1% osmium tetroxide. Fixed samples were taken through a graded ethanol and London resin (LR) medium white resin series prior to embedding in an epoxy resin. Ultrathin sections (50–100 nm) were cut using an ultramicrotome (Reichert-Jung Ultracut E) and collected on Formvar-coated copper slot grids. Initial results using post-staining with uranyl acetate and lead citrate (as used for fruits of *P. condensata*, [11]) failed to reveal a helicoidal ultrastructure. This could only be resolved when these staining stages were omitted (see electronic supplementary material, figure S1). Samples were imaged using a Hitachi H-7650 TEM equipped with an AMT XR41 digital camera.

### 2.3. Spectroscopic characterization

Reflectance spectra of the fruit surface were measured on a microscopic scale (spot size: ≈10 µm) which allowed the collection of optical signals from individual cells. The halogen lamp of the microscope served as light source in bright-field configuration. Light reflected from the sample passed back into the objective and was coupled in confocal configuration with a 100 m core optical fibre connected to a spectrometer (QE65000, Ocean Optics, 200–880 nm). The reflection spectra were normalized with respect to a silver mirror (Thorlabs). Spectra and images were collected using unpolarized illumination and a circularly polarizing filter consisting of a superachromatic quarter waveplate (B. Halle) combined with a liner polarizer (Thorlabs) for right-handed (RH) and left-handed (LH) light detection. The hyperspectral images were collected in the same configuration using an additional liquid crystal filter (CRI, Varispec) that was inserted in front of the CCD imaging chip. Images were collected with a camera and carefully normalized if recorded with different exposure times, considering also the nonlinearity of the camera response.

## 3. Results

### 3.1. Fruit anatomy

**Figure 1.**
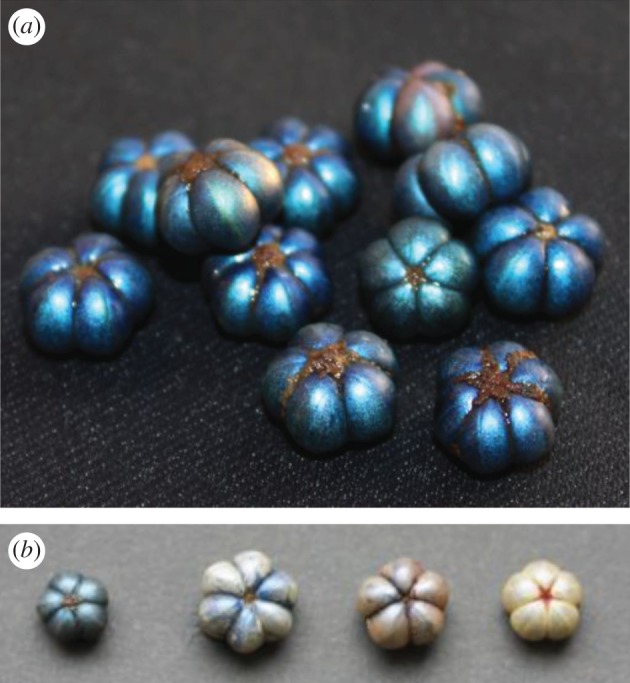
(*a*) Fresh fruits of *Margaritaria nobilis*. The intense metallic coloration of the fruits is the result of selective reflection from a helicoidal cellulose structure in the cell walls of the endocarp. (*b*) Fruits at successive stages of desiccation, from left to right: fully hydrated to dry. The average dimension of the fruits is about 1 cm.

**Figure 2.**
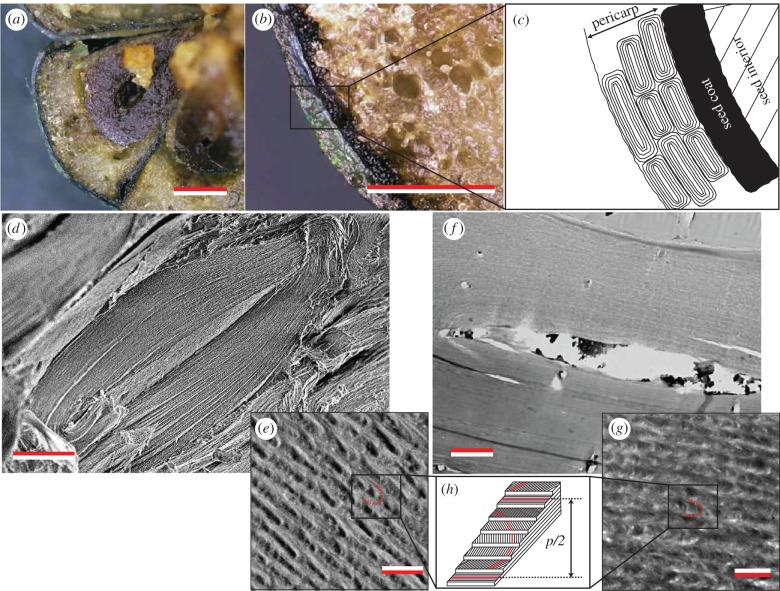
Anatomy of the *Margaritaria nobilis* fruit. (*a,b*) Transverse section of a fresh fruit shown at different magnifications. (*c*) Scheme of the fruit cross section, showing the pericarp with the multi-layered cells and the seed. (*d,e*) (*f,g*) EM transverse sections of the cell wall of a single pericarp cell, obtained with SEM and TEM, respectively, in both images a multi-layered structure can be recognized. In both cases, increasing magnification it is possible to recognize the Bouligand arch pattern (*e,g*), a clear fingerprint of a helicoidal cell-wall architecture, schematically shown in (*h*). Scale bars, 1 mm in (*a*) and 0.5 mm (*b*); 10 µm in (*d*), and 200 nm in (*e*), 3 µm in (*f*) and 500 nm in (*g*).

Each fruit of *M. nobilis* consists of several (four to six) segments, each containing a single seed (figures 1 and 2; electronic supplementary material, figure S2). The entire structure is enclosed in a pericarp that consists of two layers: an outer papery exocarp that dehisces at fruit maturity (clearly visible in figure 1 of [19]) and an endocarp consisting of three or four layers of thick-walled cells (figure 2*a–c*). The endocarp is about 1 mm thick, and the average thickness of the cell wall is about 10–15 µm. When the fruit is fresh or well hydrated, the colour of the remaining fruit is metallic blue or green. Fruits have a more pearlescent white appearance when they are completely dry (figure 1*b*).

Transverse sections of fresh fruits (figure 2*a,b*) show that the blue-green coloration of the fruits comes from the endocarp, which consists of thick-walled cells (figure 2*d–g*). When the fruit is fresh, the seeds are hydrated and adhere perfectly to the endocarp. In the dry state, the seeds shrink, and the endocarp is separated from the seeds by an air layer that prevents light absorption and therefore decreases the contrast and the saturation of the structural coloration [22].

A schematic drawing in the electronic supplementary material, figure S3, illustrates this effect and describes the mechanism of the scattering induced by the presence of the air layer. The change in macroscopic appearance of the fruit is completely reversible. By leaving the fruit in a closed environment with saturated humidity (such as in a sealed vial containing a wet tissue, not in contact with the fruit) or simply by immersing it in water, the blue coloration reappears as the fruit rehydrates. To further demonstrate that the structural colour is not lost in the dehydrated state, the micrograph in electronic supplementary material, figure S5, reveals that the colour is visible in the pericarp layer alone.

SEM and TEM cross-sectional images show the multi-layered helicoidal architecture of the cell wall structure of the endocarp cells (figure 2). At low magnification, the structure appears as a simple multilayer (figure 2*d,f*). At higher magnification and resolution (figure 2*e,g*), a Bouligand arch pattern is visible. The twist of the individual cellulose microfibrils allows to infer their organization in a helicoidal morphology.

While this helicoidal structure is readily visualized by SEM imaging, it could not be resolved by high-resolution TEM imaging of fresh material, but only in the dry state.

### 3.2. Optical characterization

**Figure 3.**
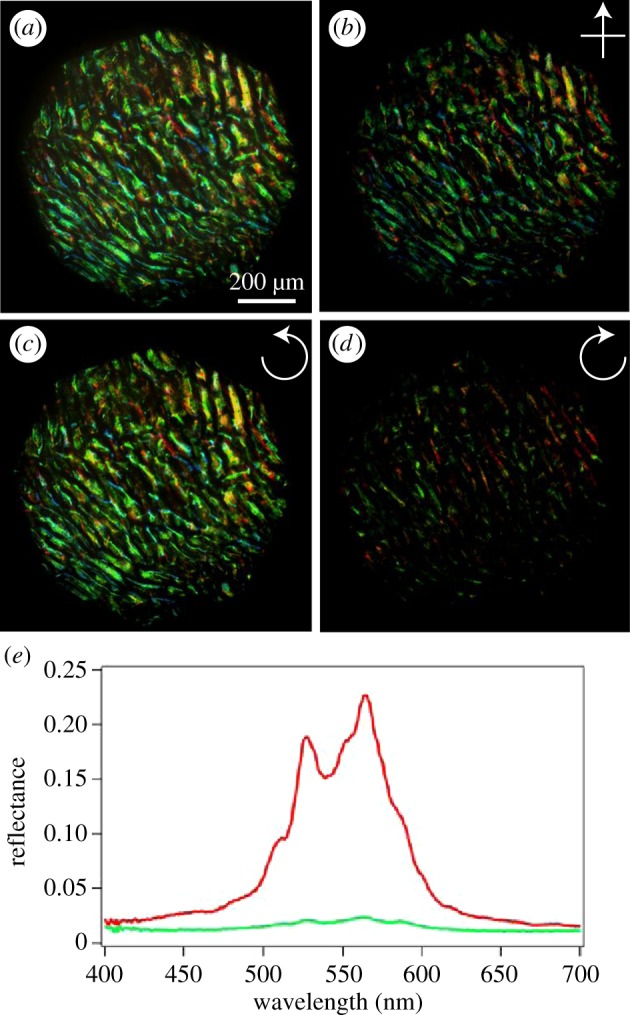
Optical response of the *Margaritaria nobilis* fruit. (*a*) Micrograph obtained using a 10× magnification objective in epi-illumination in the absence of polarization filters. Images of the same area between cross polarizers (*b*), and in left (*c*) and right (*d*) circular polarization configurations. The two spectra in (*e*) were collected from the same area in the left (red) and right (green) circular polarization channels.

Figure 3 shows the optical response of a fresh fruit illuminated at different polarization configurations. In particular, figure 3*a* shows an optical micrograph of the fruit with polarization filters in collection or illumination. In figure 3*a*, the colour reflected from the cell wall and an additional reflection that originates from the air–fruit interface can be observed. Between cross-polarizers (illuminating with polarizing light and collecting with linear polarization perpendicular to the illumination), only the reflection from the multi-layered structure is collected. The colours remain unchanged, but the image contrast sharpens (figure 3*b*).

The nature of the multilayer morphology is revealed when placing the sample between circularly polarizing filters. In this configuration, colour is observed only in the left-handed (LH) circular polarization channel (figure 3*c*), and only very little light is collected in the right-handed (RH) channel, probably scattered from inner cells tilted with respect to the surface of the fruit (figure 3*d*). It is interesting to note that all cells reflect only left-handed circularly polarized light, in contrast with cells of *P. condensata*, in which both handednesses were observed [11].

Earlier work on the structural characterization of *M. nobilis* described a concentrically layered architecture found inside individual cells [20], but the helical structure was not resolved by TEM imaging, possibly as a result of the staining issue described in §2.2.

Similar to many other examples of structural colour in nature, different cells reflect slightly different colours, as it is evident from figure 3*a*. The measured spectra therefore differ between imaged areas, even if the collection spot is smaller than the cell size, because the collected signal typically traverses a stack of several cells. Performing a detailed correlation of the reflectivity of each cell with the anatomical parameters measured from the TEM images is tricky and therefore beyond the scope of this work. However, using the extrapolated averaged pitch from TEM and the refractive index of the cellulose (*n* = 1.53), a reflection peak in the blue-green region of the spectrum is predicted, in agreement with (figure 3).

Bright-field spectra taken at the single-cell level using a 20× magnification objective are shown in figure 3*e*. In the left polarization channel (red line), several peaks are visible in the spectral region between 500 and 550 nm. In the opposite channel, only a wavelength-independent response of about 2% was recorded. This signal arises from the ubiquitous specular reflection from the interface of two media with differing refractive indices, in this case, the interface between air and the outer layer of the endocarp. Using the Fresnel equations for unpolarized illumination (equal reflectance in both polarization channels), a reflectivity of 2% is predicted for each channel, assuming a refractive index of the reflecting medium of 1.5.

**Figure 4.**
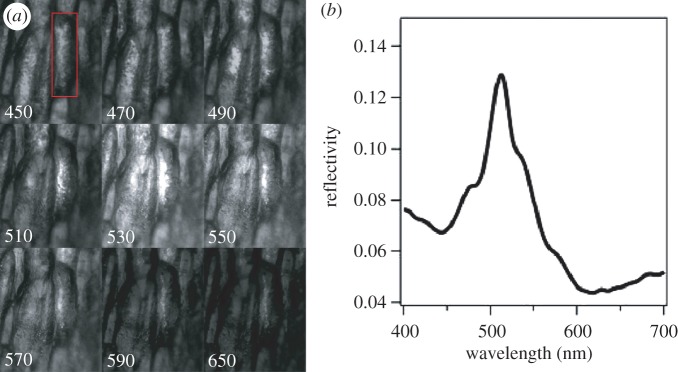
Iridescence at the single-cell level in *Margaritaria nobilis* fruit measured using hyperspectral microscopy. (*a*) Image sequence (from top to bottom and left to right) is obtained with unpolarized api-illumination using a 20× magnification objective (NA = 0.45) and a tuneable liquid crystal colour filter in front of the camera. The same area is imaged a different transmission wavelength of the liquid crystal filter, indicated in (*a*). (*b*) Spectrum measured from the cell highlighted frame in (*a*). All images in (*a*) are normalized with respect to the spectrum shown in (*b*).

In order to capture iridescence at the single-cell level, we investigated the fruit using hyperspectral microscopy. The shape of the epidermal cells of *M. nobilis* can be approximated as cylinders. As observed by Kolle *et al*. [20], when illuminating the cells with an objective with numerical aperture NA = 0.45, light is reflected from the different cells in a range of colours. This arises from the cell's curved geometry. This effect, typical of every multilayer structure with ellipsoidal or cylindrical geometry, reveals the iridescent nature of the colour, as shown in figure 4. This is however averaged out when the fruit is illuminated with diffuse light, and the iridescence disappears, leaving only an intense ‘metallic’ colour appearance. See also electronic supplementary material, figure S5 and the electronic supplementary material, movie S6.

## 4. Discussion

Our results demonstrate that the intense blue-green coloration of the fruits of *M. nobilis* is a structural effect, resulting from a helicoidal cellulose structure in the multi-layered cell walls of the pericarp. The results of our optical measurements are confirmed by high-resolution electron microscopy of the tissue showing Bouligand patterns typical of helicoidal architectures [21]. The chiral nature of the optical response of the fruit of *M. nobilis* resembles that of the fruit of *P. condensata*, except that in *M. nobilis* only left-handed polarization is reflected, whereas both LH and RH circular polarization are detected in *P. condensata* [11].

These two species are relatively distantly related among flowering plants: *P. condensata* is a commelinid monocot and *M. nobilis* is a rosid eudicot. Therefore, the detailed helicoidal cellulose structure in the fruits of these two species is clearly an example of convergent evolution of metallic fruit colour. Both species produce fruits lacking soft tissues, and therefore offer little nutritional reward to potential seed dispersers [11,19]. Although the diversity and evolution of fruit colour remains imperfectly understood [18], some studies suggest that brightly coloured non-nutritious fruits are likely to be mimetic, where the plant deceives potential dispersers such as birds by mimicking the colour of other species with fleshy nutritious fruits that grow in the same habitat [19]. This form of mimicry may allow efficient seed dispersal without the energetic cost of providing a food reward to the disperser.

Interestingly, a related example of helicoidal architecture facilitating seed dispersal occurs in some plant species with mucilaginous seed coats that adhere to passing animals. For example, in the seed coat of quince, the outer cell layers possess helicoidal thickenings that produce a slime consisting of scattered microfibrils that result from unravelling helicoidal arrays [23].

## 5. Conclusion

Our study provides a detailed correlation between the anatomy of the fruit of *M. nobilis* and its optical response. Our results demonstrate that, as in the case of *P. condensata* [11], the intense blue-green coloration of this fruit is a structural effect resulting from a helicoidal cellulose structure in the multi-layered cell walls of the pericarp. This helicoidal architecture is common, and interestingly, a related example of helicoidal architecture facilitating seed dispersal occurs in some plant species with mucilaginous seed coats that adhere to passing animals. Future studies on the internal geometry of cell walls in a diverse range of plant tissues could provide further clues concerning the construction and properties of this highly ordered and multifunctional cell-wall architecture. Even though the development of such structures in nature is not yet fully understood, material scientists have been inspired by such bright colour appearance and bioinspired photonic fibres [20] and films [24] have been produced using different strategies.

## Supplementary Material

Transmission Electron Microscop; Margaritaria nobilis fruit anatomy; Anatomy of Pericarp; Microscopy of Pericarp; Hyperspectral Microscopy

## Data accessibility

Relevant experimental data are available online in the supplementary material.
