# Supplementary material for: Structural colour from helicoidal cell-wall architecture in fruits of Margaritaria nobilis
Source: J R Soc Interface. 2016 Nov;13(124):20160645. doi: 10.1098/rsif.2016.0645 (PMC5134016; doi:10.1098/rsif.2016.0645)
Supplement: Transmission Electron Microscop; Margaritaria nobilis fruit anatomy; Anatomy of Pericarp; Microscopy of Pericarp; Hyperspectral Microscopy [file rsif20160645supp1.pdf]

Structural colour from helicoidal cell-wall architecture in fruits of *Margaritaria nobilis*

Silvia Vignolini[1], Tom Gregory[2],Mathias Kolle[4], Alfie Lethbridge[3], Edwige Moyroud[5], Ullrich Steiner[6],Beverley J. Glover[5,*], Peter Vukusic[3,*], Paula Rudall[2]

[1] *Chemistry Department, University of Cambridge, Lensfield Road , Cambridge CB2 1EW, UK*

[2] *Jodrell Laboratory, Royal Botanic Gardens Kew, Richmond, Surrey TW9 3AB, UK*

[3] *Thin Film Photonics, School of Physics, Exeter University, Exeter EX4 4QL, UK*

[4] *Massachusetts Institute of Technology 77 Massachusetts Avenue , Cambridge MA 02139-4307, USA*

[5] *Department of Plant Sciences, University of Cambridge, Downing Street, Cambridge CB2 3EA, UK*

[6] *Adolphe Merkle Institute, Chemin des Verdiers 4, 1700 Fribourg, CH*

October 23, 2016

Supplementary Material

Transmission Electron Microscopy

Imaging the helicoidal structure with high resolution with TEM can be challenging. Here in Figure S1 we show how the same fixation procedure can fail to reveal the helicoidal structure of the cell wall of *Margaritaria nobilis* fruits when as stained fresh.

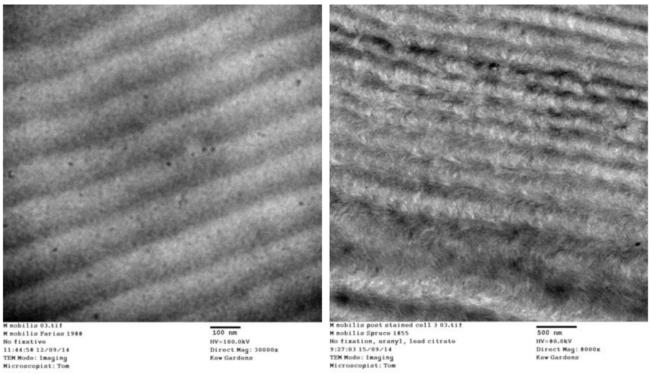

Figure S 1: TEM cross sectional images of *Margaritaria nobilis* fruit: (a) post stained cell wall of fresh *Margaritaria nobilis* specimen from herbarium compared with (b) specimen collected by Spruce in 1855.

Margaritaria nobilis fruit anatomy

As described in the main text, each fruit of *Margaritaria nobilis* consists of several (4 to 6) segments, each containing a single seed. The entire structure is enclosed in a pericarp that consists of two layers: an outer papery exocarp that dehisces at fruit maturity and an endocarp consisting of three or four layers of thick-walled cells. The endocarp is about 1mm thick, and the average thickness of the cell wall is about $10-15\mu$m. When the fruit is fresh or well hydrated the colour of the remaining fruit is metallic blue or green.

The blue-green coloration of the fruits comes from the endocarp, which consists of thick-walled cells (Figure S2, (d,e,f)). When the fruit is fresh, the seeds are hydrated and adhere perfectly to the endocarp. In the dry state, the seeds shrink, and the endocarp is separated from the seeds by an air layer that prevents light absorption and therefore decreases the contrast and the saturation of the structural coloration, see Figure S2. Transverse sections of fresh fruits are shown in (Figure S3(a,b,c)) for different magnifications and methods, as described in the caption.

Hyperspectral Microscopy

Hyperspectral microscopy allows to visualise how the maximum of reflectivity is spatially distributed on the surface. Due to the fact that the shape of the helicoidal structure is not flat but is "wrap around" the cell the hyperspectral imaging can show how the colour shift in function of the position in the same cell.

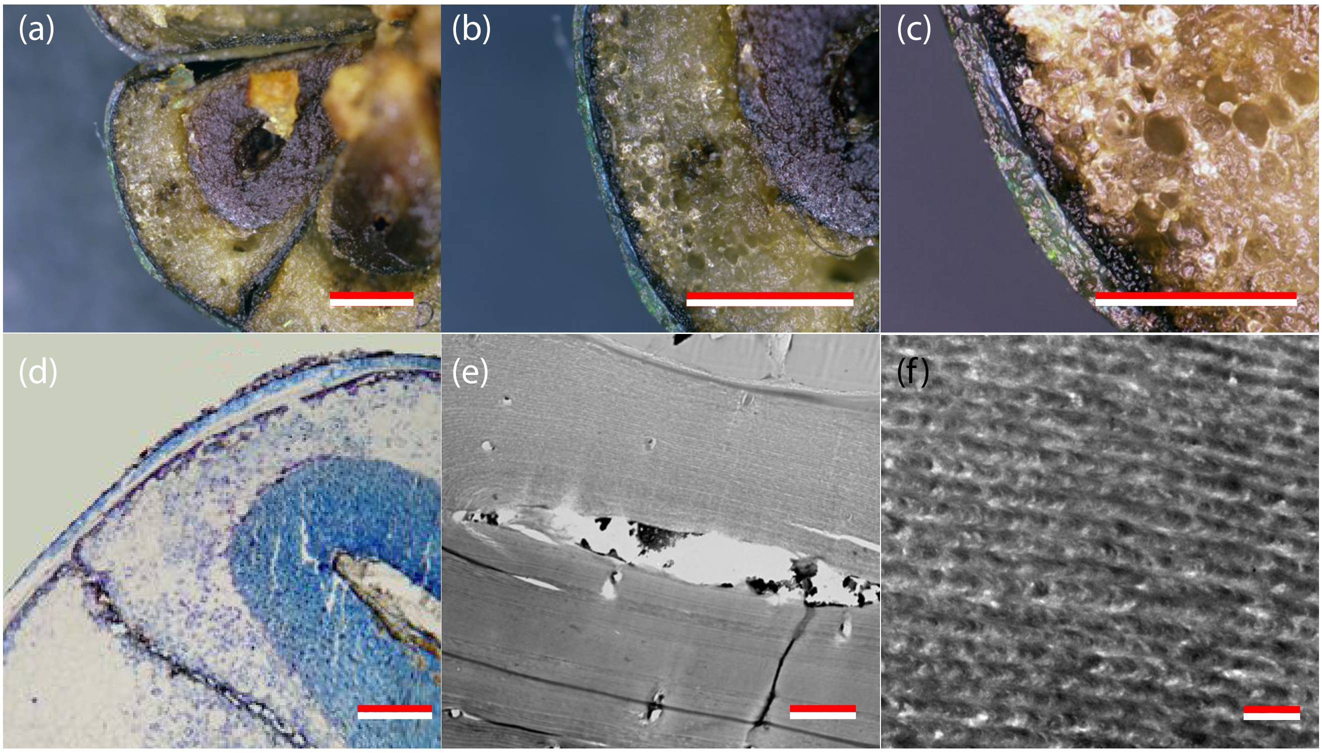

Figure S 2: Anatomy of *Margaritaria nobilis* fruit. *(a,b,c)* Transverse section of a fresh fruit shown at different magnifications. *(d)* Transverse section of fruit stained with toluidine blue. The pericarp is the light blue outermost layer. *(e,f)* EM transverse sections of the cell wall of a single pericarp cell, with multilayered structure visible in *(e)*, and Bouligand arch pattern, the fingerprint of helicoidal cell-wall architecture, visible at higher magnification in *(f)*. Scale bars: 1mm in *(a)* and *(b)*, 0.5 mm in *(c)*, 200μm in *(d)*, and 4μm in *(e)* and 0.5μm in *(f)*.

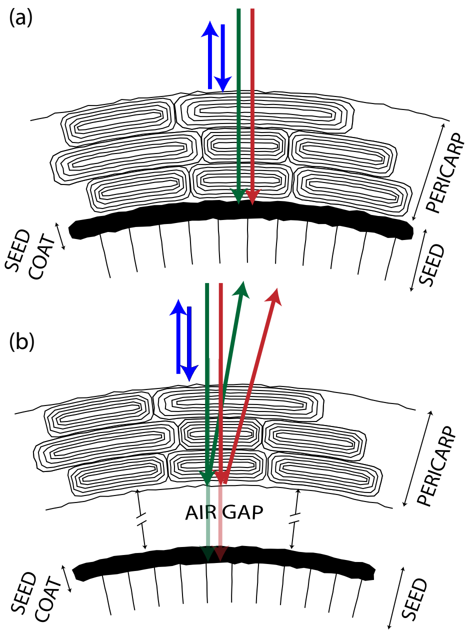

Figure S 3: Scheme of the fruit cross section, showing the pericarp with the multilayered cells and the seed in wet (a) and dry (b) state. The change of the appearance can be understood in terms of scattering. When the fruit is wet the seeds adhere to the pericarp and the light that is not reflected by the helicoidal cells in the pericarp layer is absorbed by dark-pigmented cell in the the seed coat, making the cell appear blue macroscopically. On the other hand, when the fruit is dry the seed are well separated by the pericarp and the presence of a second interface also partially scatters all the wavelength of the visible light and give the whitish appearance pearlescent appearance.

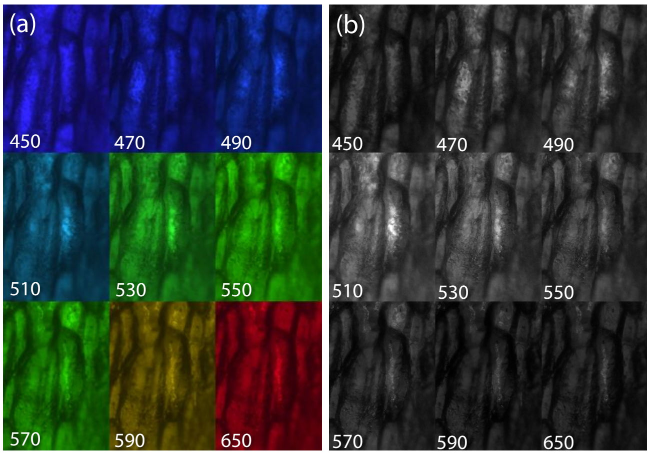

Figure S 5: Raw data on the iridescence on the single cell level of *Margaritaria nobilis* fruit obtained with hyperspectral microscopy as shown in Figure 4*(a)*. Set of micrograph pictures obtained with a 20× magnification objective (NA = 0.45) in epi-illumination with the liquid crystal filter in real colour. Processed image as reported in Figure 4 *(a)* where the averaged intensity is normalised with respect the spectrum reported Figure 4 *(b)*.

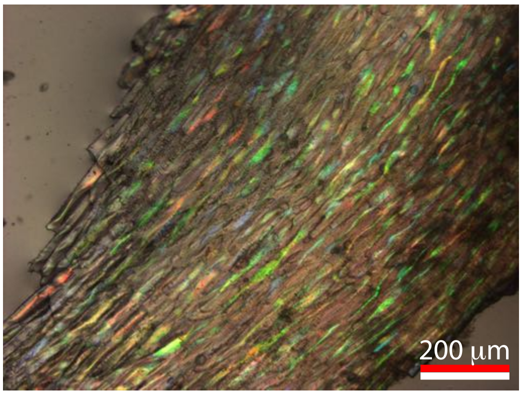

Figure S 4: Optical Micrograph picture obtained using a 5× magnification objective in epi-illumination without any polarisation filter, of the pericarp layer alone on a glass slide.

Figure S 6: The Movie reports in each frame a micrograph picture obtained with a 20× magnification objective (NA = 0.45) in epi-illumination with the liquid crystal filter in real colour. In each frame the bandwidth of the filter is charged at step of 10 nm form 400 to 610.
